# Supplementary material for: Mapping global evidence on strategies and interventions in neurotrauma and road traffic collisions prevention: a scoping review protocol
Source: BMJ Open. 2019 Nov 12;9(11):e031517. doi: 10.1136/bmjopen-2019-031517 (PMC6858136; doi:10.1136/bmjopen-2019-031517)
Supplement: Author's manuscript [file bmjopen-2019-031517.draft_revisions.pdf]

BMJ Open is committed to open peer review. As part of this commitment we make the peer review history of every article we publish publicly available.

When an article is published we post the peer reviewers' comments and the authors' responses online. We also post the versions of the paper that were used during peer review. These are the versions that the peer review comments apply to.

The versions of the paper that follow are the versions that were submitted during the peer review process. They are not the versions of record or the final published versions. They should not be cited or distributed as the published version of this manuscript.

BMJ Open is an open access journal and the full, final, typeset and author-corrected version of record of the manuscript is available on our site with no access controls, subscription charges or pay-per-view fees (http://bmjopen.bmj.com).

If you have any questions on BMJ Open's open peer review process please email info.bmjopen@bmj.com

BMJ Open

A scoping review protocol to map the evidence on strategies and interventions in neurotrauma and road traffic collisions prevention globally

Journal:	*BMJ Open*
Manuscript ID	bmjopen-2019-031517
Article Type:	Protocol
Date Submitted by the Author:	09-May-2019
Complete List of Authors:	M Selveindran, Santhani; University of Cambridge Institute of Public Health; University of Cambridge, NIHR Global Health Research Group on Neurotrauma<br>Khan, Muhammad; Northwest School of Medicine and Northwest General Hospital and Research Centre; University of Cambridge, NIHR Global Health Research Group on Neurotrauma<br>Simadibrata, Daniel; University of Indonesia; Newcastle University, Faculty of Medical Sciences<br>Hutchinson, Peter; Addenbrooke's Hospital, Department of Clinical Neurosciences; University of Cambridge, NIHR Global Health Research Group on Neurotrauma<br>Brayne, Carol; University of Cambridge Institute of Public Health; University of Cambridge, NIHR Global Health Research Group on Neurotrauma<br>Hill, Christine; University of Cambridge Institute of Public Health; University of Cambridge, NIHR Global Health Research Group on Neurotrauma<br>Kolias, Angelos; Addenbrooke's Hospital, Department of Clinical Neurosciences; University of Cambridge, NIHR Global Health Research Group on Neurotrauma<br>Joannides, Alexis; Addenbrooke's Hospital, Department of Clinical Neurosciences; University of Cambridge, NIHR Global Health Research Group on Neurotrauma<br>Servadei, Franco; Humanitas University and Research Hospital, Department of Neurosurgery; World Federation of Neurosurgical Societies<br>Rubiano Escobar, Andres ; Universidad El Bosque, Department of Neurosurgery; University of Cambridge, NIHR Global Health Research Group on Neurotrauma<br>Shabani, Hamisi; Muhimbili Orthopaedic Institute and Muhimbili University College of Allied Health Sciences; University of Cambridge, NIHR Global Health Research Group on Neurotrauma
Keywords:	Neurotrauma, Traffic accidents, Accident prevention, Preventative interventions, Preventative strategies

SCHOLARONE™
Manuscripts

TITLE: A scoping review protocol to map the evidence on strategies and interventions in neurotrauma and road traffic collisions prevention globally

Santhani M Selveindran [1,2], Muhammad Mukhtar Khan [2,3], Daniel M Simadibrata [4,5], Peter JA Hutchinson [2,6], Carol Brayne [1,2], Christine Hill [1,2,7], Angelos Kolias [2,6], Alexis Joannides [2,6], Franco Servadei [8,9], Andres M Rubiano Escobar [2,10], Hamisi K Shabani [2,11].

1. *Cambridge Institute of Public Health, University of Cambridge, Cambridge, UK.*
2. *NIHR Global Health Research Group on Neurotrauma, University of Cambridge, Cambridge, UK.*
3. *Northwest School of Medicine and Northwest General Hospital & Research Center, Peshawar, Pakistan.*
4. *University of Indonesia, Depok, Jawa Barat, Indonesia.*
5. *Faculty of Medical Sciences, Newcastle University, Newcastle upon Tyne, UK.*
6. *Division of Neurosurgery, Department of Clinical Neurosciences, Addenbrooke's Hospital and University of Cambridge, Cambridge, UK.*
7. *Collaboration for Leadership in Applied Health Research and Care East of England (CLAHRC), Cambridge, UK.*
8. *Department of Neurosurgery, Humanitas University and Research Hospital, Milan, Italy*
9. *World Federation of Neurosurgical Societies, Nyon, Switzerland*
10. *Neuroscience Institute, El Bosque University, Bogotá, Colombia.*
11. *Neurological Surgery Unit, Muhimbili Orthopedic Institute and Muhimbili University College of Allied Health Sciences, Dar es Salaam, Tanzania.*

Correspondance to: Dr Santhani M Selveindran, Cambridge Institute of Public Health, University of Cambridge School of Clinical Medicine, Forvie Site, Cambridge Biomedical Campus, Cambridge CB2 0SR, UK. Email: ss2604@medschl.cam.ac.uk. Tel: +44122330301.

Keywords: *Neurotrauma, Traffic accidents, Accident prevention, Preventative interventions, Preventative strategies*

Word count: 3112

For peer review only - http://bmjopen.bmj.com/site/about/guidelines.xhtml

ABSTRACT

Introduction: Neurotrauma is an important global health problem. This 'silent epidemic' is a major cause of death and disability in adolescents and young adults, with significant societal and economic impacts. Globally, the largest cause of neurotrauma is road traffic collisions (RTCs). Neurotrauma and RTCs are largely preventable, and many preventative strategies and interventions have been established and implemented over the last decades, particularly in high income countries. However, these strategies may not be applicable globally, due to variations in environment, resources, population, culture and infrastructure. This paper outlines the protocol for a scoping review which seeks not only to map the evidence on strategies and interventions in neurotrauma and RTC prevention globally, but also to ascertain contextual factors that affect or influence their implementation.

Methods and analysis: This scoping review will use the established methodology by Arksey and O'Malley. Eligible studies will be identified from electronic databases and grey literature sources. We will also carry out bibliographic and citation searching of the eligible studies. A two-stage screening process which involves screening of titles and abstracts, followed by full-text screening will be used to determine eligible studies, which will undergo data abstraction using a customized, piloted data-extraction sheet. The extracted data will be presented using evidence mapping and a narrative summary.

Ethics and dissemination: Ethics approval is not required for this scoping review. The final review will be submitted for publication to a scientific journal, and results will be presented at appropriate conferences, workshops and meetings. This scoping review is also the first step in a multi-step public health research project on the global prevention of neurotrauma, and forms part of a scoping exercise to glean contextual issues and research priorities. The review will be part of the report that will be submitted to the funders and relevant stakeholders.

ARTICLE SUMMARY

STRENGTHS AND LIMITATIONS OF THIS STUDY

- This will be the first scoping review to identify and quantify the evidence on all types of preventative strategies and interventions for primary, secondary and tertiary prevention of neurotrauma, using all types of literature in both high and low to middle income countries.
- The scoping review utilizes a well-established, rigorous scoping review methodology with a systematic, iterative search strategy.
- The literature search will be comprehensive, encompasses electronic databases with peer-reviewed literature and conference proceedings, as well as a wide range of grey literature sources including government and non-governmental websites.
- A limitation of this review is the language restriction to the English, which may result in the potential to miss relevant articles from low and middle income countries, especially in the grey literature.

For peer review only

For peer review only - http://bmjopen.bmj.com/site/about/guidelines.xhtml

BACKGROUND

Neurotrauma is an important global health problem (1-2). Previous studies and systematic reviews, including one from the World Health Organization (WHO) estimate that the annual global incidence of neurotrauma is approximately 500 to 800 per 100,000 (3-4). This ‘silent epidemic’ continues to increase and is predicted to surpass many diseases as a major cause of death and disability (5). Indeed, WHO Global Burden of Disease estimates indicate that neurotrauma accounts for about 11.8 percent of total global disability-adjusted life years (DALYs) (3).

Although neurotrauma typically refers to injury to the brain and/or spinal cord, for this review, neurotrauma will focus on injuries to the head alone.

Globally, the largest cause of neurotrauma is road traffic collisions (RTCs) (3, 5). RTCs are the eighth leading cause of death in the world, and recent WHO analyses estimate that this could become the fifth leading cause of death by 2030 (6). RTCs are particularly common in low and middle income countries (LMICs) as a result of rapid urbanization and motorization with few or no safeguarding measures (6-8).

Irrespective of cause, neurotrauma poses major medical, public health and societal problems worldwide due to the impact it has on individuals, families, communities, governments and healthcare systems. (9-13).

Despite some country-to-country variability, many studies have shown that neurotrauma is more common in children and young adults (5, 10, 14). Neurotrauma is a major cause of death and the leading cause of disability in those under forty years of age, and is predicted to be the 3rd leading cause of premature death across all ages by 2020 (4,14). Generally, there is a bimodal age-specific neurotrauma incidence with peaks in childhood (0-10 years) and late adolescence/early adulthood (15-36 years) (10, 14). In terms of gender, the incidence of neurotrauma is consistently higher in males with ratios ranging from 1.6 to 6.5, particularly in adolescence and early adulthood (4-5, 14).

The demographic impact of neurotrauma in itself has societal and economic implications. As neurotrauma death is highest in males in the age-group where most would be the breadwinners within their families, this can result in financial loss or burden (9). In those who survive, a large proportion would require prolonged hospitalisation, long rehabilitation and suffer long-term disabilities (15). Deficits associated with neurotrauma include cognitive (impaired attention, poor executive functioning, poor decision-making), physical (motor functioning), behavioural (aggressive behaviour, impulsivity) and emotional (depression, anxiety) (16-18). All this would not only affect their ability to work, but to care for themselves and to participate in society, impacting the lives of relatives and the community around them (9, 19-20). There would be loss of quality of life (intangible costs), not only for the sufferer, but also for their caregivers (12, 18).

Economic losses do not only affect families, but also have impact on countries and the world as a whole. The global economic cost of neurotrauma is estimated to be in the region of USD 400 billion annually, where most of the aggregate cost of neurotrauma takes the form of indirect costs, resulting from the adverse effect on people’s ability to work (loss of productivity) (9, 12, 21). The direct costs, which refer to all resources consumed within the health-care sector as a result of neurotrauma, pose a burden on healthcare systems (9, 22).

The management of neurotrauma encompasses pre-hospital care (lasting for minutes to hours), in-hospital (hours to weeks) and post-acute care (weeks to years) (12, 14, 23-24). Pre-hospital care involves the correct assessment and efficient treatment at the site of injury or during transfer, and

For peer review only - http://bmjopen.bmj.com/site/about/guidelines.xhtml

the prompt transport to a formal, appropriately equipped healthcare facility (12, 24). In-hospital care would be the surgical and non-surgical treatment of neurotrauma, including imaging, neuro-monitoring and intensive care, whereas post-acute care refers to any form of rehabilitative interventions to enable and empower patients to have an increased quality of life, as neurotrauma is a life-long disorder (5, 23-24).

The lifetime costs of medical treatment for neurotrauma is said to range from USD 600,000 to USD 1.8 million per case and are almost always borne by the healthcare system (21-22). In addition to financial constraint, the high burden of neurotrauma may be disproportionate to the resources available for managing the patient load, particularly in LMICs where there are deficiencies in facilities and manpower (5, 14, 25).

Having said all this, neurotrauma prevention would be more beneficial in that it would not only save lives, but reduce prevalence of disabilities and save costs within and without the health-care system (12). Neurotrauma prevention is not only limited to measures which target injury occurrence (primary prevention), but also involves providing adequate medical response to manage and minimise harm following an injury (secondary prevention), and mitigating the sequelae and reducing consequent disability (tertiary prevention) (12, 26-27). These can be applied at societal, community, household and individual levels (12, 28).

Many high-income countries (HICs) have implemented multiple intervention strategies and projects that have contributed significantly to the reduction of neurotrauma (29). These interventions range from adapting the environment, legislation, safety education and skills training, to strengthening post-trauma response systems and improving access to acute and post-acute care (12, 23, 30-32). However, these strategies are not often seen in LMICs due to differences in environment, resources, population, culture and infrastructure (33-35). It is therefore necessary that interventions and strategies match contexts, in order to effectively reduce the burden of neurotrauma, not only in LMICs but globally.

To that end, the objective of this scoping review is to identify and quantify the breadth of evidence on neurotrauma preventative strategies and interventions, provide a descriptive overview of what these are, where they are implemented, and to ascertain contextual factors that influence their success (or not).This in turn will be used to inform policy and practice in the area of neurotrauma prevention. As RTCs are a major cause of neurotrauma, this review will delve into RTC prevention, in addition to neurotrauma prevention as a whole. For this review, RTCs will be defined as a collision or incident involving at least one motorized or unmotorized (i.e. pedestrian, cyclist) vehicle in motion, on a road to which the public has right of access (36).

This methodology is appropriate for reviewing a large body of literature to generate an overview of the evidence on this subject, summarise the results and identify research gaps (37). To date, several reviews have been carried out to identify, examine, and study effectiveness of specific preventative strategies in particular regions or countries (29, 33, 38-49). There is also a scoping review on interventions to reduce RTCs, but this was limited to the African continent, and another one specifically on physiotherapy after neurotrauma (6, 50). As such, the value of this scoping review is that it will include all types of evidence on all types of preventative strategies and interventions in both HICs and LMICs.

For peer review only - http://bmjopen.bmj.com/site/about/guidelines.xhtml

For peer review only

METHODS

Protocol design

This scoping review is informed by the framework proposed by Arksey and O'Malley (2005). This is a six step framework that includes:

1. Identifying the research question
2. Identifying relevant studies
3. Study selection
4. Charting the data
5. Collating, summarizing, and reporting the results
6. Consulting and translating knowledge

As this scoping review is part of a wider scoping exercise that includes stakeholder consultation, stage 6 will not be carried out.

Stage 1: Identifying the research question

Being part of a wider scoping exercise that seeks to understand contextual issues relating to neurotrauma and RTC prevention, the following research questions were identified:

1. What types of strategies or interventions to prevent neurotrauma or RTCs in LMICs are available in the literature?
2. What types of strategies or interventions to prevent neurotrauma or RTCs in HICs are available in the literature?
3. In what settings are these strategies or interventions carried out (i.e school-based/community-based)?
4. What are the contextual factors that can affect or influence the implementation of these strategies or interventions?

Stage 2: Identifying relevant studies

In this stage, we will identify the criteria that will be used to select studies that will be included in the review. The following eligibility criteria will be utilized for study selection:

Study population: Adults and children, where children will be taken as those below the age of 18 years.

Concept: Preventative strategies or interventions for neurotrauma or RTCs. For this review, this would include primary prevention, which refers to any measures that eliminate neurotrauma or RTCs; secondary prevention, which for this review will refer to pre-hospital care systems and any interventions delivered as part of this care; and tertiary prevention, which for this review will be any form of rehabilitative interventions for neurotrauma patients. We will include publications which describe established preventative intervention(s). Studies reporting on intervention outcomes which relate to neurotrauma and/or RTC prevention, or on factors influencing implementation will also be included. For RTC prevention, we will include studies on strategies or interventions that prevent crashes and those that prevent neurotrauma, should a crash occur.

We will exclude the following studies: Papers on interventions that do not specifically prevent neurotrauma and/or RTCs, studies where interventions are ill-defined, publications describing potential interventions or opinions on an intervention, studies on interventions without any specific context, papers describing only the prevalence of intervention utilization without measuring

For peer review only - http://bmjopen.bmj.com/site/about/guidelines.xhtml

outcomes or exploring factors influencing utilization, and publications discussing in-hospital management or care of neurotrauma patients.

For publications on rehabilitative interventions (tertiary prevention), only articles discussing specific interventions or strategies that address cognitive, physical, behavioural and emotional rehabilitation will be included. Articles including pharmacological interventions will be excluded.

Context: The preventative strategies or interventions would be carried out or delivered in any country (LMIC or HIC) and in any setting (i.e. community or health-care facility)

Language: Only studies written in English will be included.

Time frame: Searches will begin from 1974 onwards, which was when the World Health Assembly adopted Resolution WHA 27.59, calling for Member States to address the major public health problem of RTCs (31). For databases established after 1974, searches will commence from their inception.

Types of studies: As a scoping review is designed to cover a wide spectrum of literature, there will be no restrictions as to the types of studies that will be included in this review.

The following electronic databases will be searched:

1. MEDLINE (Ovid)
2. EMBASE (Ovid)
3. CINAHL (EBSCO host)
4. Global Health (EBSCO host)
5. Cochrane Database of Systematic Reviews

An academic librarian was consulted and gave suggestions on key concepts, and text-word and Medical Subject Headings searching. The initial search terms were determined by the main author where the concepts 'Neurotrauma', 'Road traffic collisions' and 'Prevention' were each expressed with a list of synonyms. This was supplemented with keywords and phrases from relevant articles retrieved from an exploratory search of MEDLINE and Google Scholar (see online supplementary material, **APPENDIX**, which details the search strategy developed for MEDLINE). This will be utilized in the other electronic databases, where the search terms will be adapted and modified based on the requirement of the individual database. As scoping review searches are quite iterative, additional search terms may be identified and incorporated into the search strategy.

We will also search "grey" literature to identify any non-indexed papers. This would include web-based data sources such as Open Grey, Prevention Information and Evidence Library, The Grey Literature Report and The National Institute for Health and Care Excellence (NICE).

Where possible, we will search for unpublished studies from websites of governmental, non-governmental or patient-based organizations engaged in neurotrauma or RTC prevention (i.e. Global Alliance of NGOs for Road Safety).

Bibliographies of eligible studies will also be examined to identify any original studies not obtained through the searches. Additionally, citation searching of these studies, using Google Scholar will also be carried out.

Search results will be imported to a bibliographic manager which will be used to store references and remove duplicates. Where necessary, duplicates will also be removed manually.

For peer review only - http://bmjopen.bmj.com/site/about/guidelines.xhtml

Stage 3: Study selection

The screening for potentially eligible publications will follow a two-stage process:

Stage 1: The titles and abstract of potentially eligible studies will be screened against selection criteria, and will be categorized as 'include', 'exclude' or 'uncertain'.

Stage 2: Full text articles will be sourced for those publications under the 'include' and 'uncertain' categories. The full-text articles will be assessed against the selection criteria and the reason for exclusion will be documented.

This process will be carried out by two reviewers independently. Any disagreements regarding publication inclusion will be resolved through discussion between the two reviewers or third party adjudication.

The process of study selection will be reported using the Preferred Reporting Items for Systematic Reviews and Meta-Analyses (PRISMA) flow chart.

Stage 4: Charting the data

The extraction of data for a scoping review is known as 'charting the data' and should present information about the study that aligns to the objective and questions of the review.

Appropriate data from the eligible studies will be extracted using a customized data-extraction sheet. This will be in a tabular form and will be piloted on several studies to ensure all relevant results are extracted, pertinent to the research questions. If necessary, the categories will be modified and the extraction sheet revised.

In line with the research questions for this review, the following data will be extracted:

Publication detail:

- Citation
- Publication type (published/unpublished)
- Publication source (journal/website)
- Country of origin
- Funding source

Study characteristics:

- Study design/type
- Aim(s)/objective(s) of study
- Study location/setting

If Systematic review/meta-analyses:

- Number of studies included
- Years of publication of included studies
- Countries where included studies were conducted

For peer review only - http://bmjopen.bmj.com/site/about/guidelines.xhtml

For peer review only

Participant characteristics:

- Age/age-range
- Gender
- Ethnicity
- Occupation/socio-economic status
- Number of participants

Preventative strategy/intervention

- Description of intervention
- Delivery of intervention (how and by whom)
- Setting of intervention
- Length and intensity of intervention

Reported outcome(s)

Factors influencing outcome(s)

The charting of data is an iterative process and the extraction fields may change depending on the included studies.

Stage 5: Collating, summarizing and reporting results

The data collected will be presented using two strategies:

1. Evidence mapping to provide a comprehensive and concise descriptive map of the breadth of research on neurotrauma or RTC prevention. The extracted data will be categorized based on the year of publication, study design, geographic region, participants, type of prevention (primary, secondary, tertiary) and type of intervention. Data will be presented in tabular and graphical forms.

2. A narrative summary describing the included studies and how the results relate to the review objective and research questions.

The exact reporting format cannot be determined until the data is charted, which would depend on the literature found. If possible, a specific framework will be sought to categorize and map the findings.

There will be no assessment of quality as it is beyond the scope of the review.

For peer review only

Patient and Public Involvement

Although patients and public are not involved in the design and conduct of this scoping review, it forms part of a larger scoping exercise which seeks to gather contextual issues and align research priorities. The review will be used to guide questioning during the stakeholder consultation, which is the second component of this exercise. Stakeholders will include policy makers, law enforcement officials, healthcare staff, and members of patient and advocacy groups, who could have been neurotrauma patients. Comparisons will be made between findings from this review and the stakeholder consultation.

This review will also form part of the report on the scoping exercise that will be sent for publication and shared with relevant stakeholders and research funders. We will aim to utilize governmental and non-governmental websites and facilities to disseminate data to patients and members of the public.

CONCLUSION

This article presents a protocol for a scoping review, which is a comprehensive, rigorous and transparent methodology. This review, which includes both peer reviewed and grey literature, will contribute to research in the area of neurotrauma and RTC prevention by identifying the breadth of preventative strategies and interventions, their contexts and factors influencing success. Through this research, policy and practice can be informed, leading to the development and implementation of evidence-based, context-appropriate, meaningful and feasible interventions in resource constrained settings. This review also allows for discerning gaps in knowledge, and to provide recommendations for future research.

This scoping review is the first step in a multistep research project which aims to study the epidemiology of RTCs in LMICs and to understand factors amenable to neurotrauma prevention in these countries.

ETHICS AND DISSEMINATION

As the scoping review methodology entails collecting, reviewing and synthesising material from publicly available publications, no ethical approval is necessary. The review will be submitted for publication to a scientific journal. Where possible, results will be presented at appropriate conferences, workshops and meetings, both nationally and internationally.

For peer review only

STATEMENTS

Acknowledgements: The authors thank Dr Veronica Phillips for giving advice and feedback on the search strategy. They also thank Dr Sarah Kelly for providing advice on the research questions and overall conduct of the scoping review.

Authors' contributions: SMS conceived the idea of a scoping review, developed the research questions and methods, and contributed substantially to drafting and editing this protocol. MMK and DMS helped conceptualise the study inclusion and exclusion criteria. PJAH, AK, CB, CH, AJ, FS, AMRE and HKS gave guidance on study design and provided advisory contributions to the development of this protocol. All authors give approval to the publishing of the protocol manuscript.

Funding: This research was commissioned by the National Institute of Health Research using Official Development Assistance (ODA) funding (Grant number: RG89187). The views expressed in this publication are those of the author(s) and not necessarily those of the NHS, National Institute for Health Research or the Department of Health.

Competing interests: None

Provenance and peer review: Not commissioned; externally peer reviewed

Open Access: This is an Open Access article distributed in accordance with the Creative Commons Attribution Non Commercial (CC BY-NC 4.0) license which permits others to distribute, remix, adapt, build upon this work non-commercially, and license their derivative works on different terms, provided the original work is properly cited and the use in non-commercial. See: htto://creativecommons.org/licenses/by-nc/4.0/

REFERENCES

1. Green SE, Bosch M, McKenzie JE, O'Connor DA, Tavender EJ, Bragge P et al. Improving the care of people with traumatic brain injury through the Neurotrauma Evidence Translation (NET) program: protocol for a program of research. Implementation Science. 2012; 7 (24). Available from http://www.implementationscience.com/content/7/1/74
2. GBD 2016 Traumatic Brain Injury and Spinal Cord Injury Collaborators. Global, regional, and national burden of traumatic brain injury and spinal cord injury, 1990-2016: a systematic analysis for the Global Burden of Disease Study 2016. Lancet Neurology. 2019; 18: 56-87.
3. Dewan M, Rattani A, Gupta S, Baticulon RE, Hung Y, Punchak M et al. Estimating the global incidence of traumatic brain injury. Journal of Neurosurgery. April 27, 2018. **DOI:** https://doi.org/10.3171/2017.10.JNS17352.
4. Feigin VL, Barker-Collon S, Krishnamurthi R, Theadom A, Starkey N. Epidemiology of ischaemic stroke and traumatic brain injury. Best Practice and Research Clinical Anaesthesiology. 2010; 24: 485-494.
5. Hyder AA, Wunderlich CA, Puvanachandra P, Gururaj G and Kobusingye OC. The impact of traumatic brain injuries: A global perspective. NeuroRehabilitation. 2007; 22(5): 341-353.
6. Bonnet E, Lechat L, Ridde V. What interventions are required to reduce road traffic injuries in Africa? A scoping review of the literature. PLos One. 2018; 13 (11): e208195.
7. Staton C, Vissoci J, Gong E, Toomey N, Wafula R, Amdelgadir J et al. Road Traffic Injury Prevention Initiatives: A Systematic Review and Metasummary of Effectiveness in Low and Middle Income Countries. PloS ONE. 2015; 11(1): e0144971.

For peer review only - http://bmjopen.bmj.com/site/about/guidelines.xhtml

8. Johnson WD, Griswold DP. Traumatic Brain Injury: a global challenge. The Lancet Neurology Commission. 2017; 16: 948-950.
9. Majdan M, Plamcikova D, Brazinova A, Rusnak M, Nieboer D, Feigin V et al. Epidemiology of traumatic brain injuries in Europe: cross-sectional analysis. Lancet Public Health. 2016; 1: e76-83.
10. Saatian MR, Ahmadpoor J, Mohammadi Y, Mazloumi E. Epidemiology and Pattern of Traumatic Brain Injury in a Developing Country, Regional Trauma Center. Bulletin of Emergency Trauma. 2018; 6 (1): 45-53.
11. Wilson MH. Traumatic Brain Injury: an underappreciated public health issue. The Lancet. 2016; 1(2): pe44.
12. Maas AIR, Menon DK, Adelson PD, Andelic N, Bell MJ. Belli A, Bragge P, Brazinova A et al. Traumatic brain injury: integrated approaches to improve prevention, clinical care, and research. The Lancet Neurology Commission. 2017; 16: 987-1048.
13. Wright DW, Kellermen A, McGuire LC, Chen B. Popovic T. CDC Grand Rounds: Reducing Severe Traumatic Brain Injury in the United States. Morbidity and Mortality Weekly Report. 2013; 62 (27): 549-552.
14. Iaccariono C, Carretta A, Nocolosi F, Morselli C. Epidemiology of severe traumatic brain injury. Journal of Neurosurgical Sciences. 2018; 62 (5): 535-541.
15. Stocchetti N, Zainer ER. Chronic impact of traumatic brain injury on outcome and quality of life: a narrative review. Critical care. 2016; 20 (148). DOI.10.1186/s13054-016-1318-1.
16. Rubiano AM, Carney N, Chestnut R, Puyana JC. Global neurotrauma research challenges and opportunities. Nature. 2015; 527: S193-S197.
17. Ponsford J, Spitz G. MERRC-TAC Linkage Report: identifying different patterns of outcome and their predictors. 2016. Monash-Epworth Rehabilitation Research Centre, Monash University, Australia: Research report 150-0816-R02.
18. Connolly D, O'Dowd T. The Impact of the Different Disabilities arising from Head Injury on the Primary Caregiver. British Journal of Occupational Therapy. 64 (1): 41-45.
19. Access Economics Pty Limited. The economic cost of spinal cord injury and traumatic brain injury in Australia. 2009. The Victorian Neurotrauma Initiative, Victoria, Australia.
20. Juengst SB, Arenth PM, Raina KD, McCue M, Skidmore ER. Affective State and Community Integration after Traumatic Brain Injury. Americal Journal of Physiotherapy and Medical Rehabilitation. 2014; 93 (12): 1086-1094.
21. Parsonage M. The costs of traumatic brain injury. February 2017. Centre for Mental Health. London, United Kingdom.
22. Fu TS, Jing R, McFaull SR, Cusimano MD. Health and Economic Burden of Traumatic Brain Injury in the Emergency Department. The Canadian Journal of Neurological Sciences Inc. 2016; 43 (2). 238-247.
23. Cnossen MC, Lingsma HF, Tenovuo O, Maas AIR, Menon D et al. Rehabilitation After Traumatic Brain Injury: A Survey in 70 European Neurotrauma Centres Participating in the CENTER-TBI Study. Journal of Rehabilitation Medicine. 2017; 49: 395-401.
24. Dash HH, Chavali S. Management of traumatic brain injury patients. Korean Journal of Anesthesiology. 2018; 71(1): 12-21.
25. Zia N, Mehmood A, Namaganda RH, Ssenyonjo H, Kobusingye O, Hyder AA. Causes and outcomes of traumatic brain injuries in Uganda: analysis from a pilot hospital registry. Trauma Surgery and Acute Care Open. 2019; 4: e000259.
26. Hoz S, Moscote-Salazar LR. Prevention of Neurotrauma: An Evolving Matter. Journal of Neurosciences in Rural Practice. 2017; 8 (Suppl 1): S141-S143.

For peer review only - http://bmjopen.bmj.com/site/about/guidelines.xhtml

27. Gururaj G, Kolliri SVR, Chandramouli BA, Subbakirshna DK, Kraus JF. Traumatic Brain Injury. National Institute of Mental Health and Neuro Sciences. 2005. Publication no.61. Bangalore.
28. Wright DW, Kellermen A, McGuire LC, Chen B. Popovic T. CDC Grand Rounds: Reducing Severe Traumatic Brain Injury in the United States. Morbidity and Mortality Weekly Report. 2013; 62 (27): 549-552.
29. Staton C, Vissoci J, Gong E, Toomey N, Wafula R, Amdelgadir J et al. Road Traffic Injury Prevention Initiatives: A Systematic Review and Metasummary of Effectiveness in Low and Middle Income Countries. PloS ONE. 2015; 11(1): e0144971.
30. Wood S, Bellis MA, Watkins S. Road traffic accidents: A review of evidence for prevention from the UK focal point for violence and injury prevention. 2010. Centre for Public Health, Liverpool.
31. World Health Organization (WHO). World Report on Road Traffic Injury Prevention. 2004 WHO. Geneva.
32. Roudsari BS, Nathens AB, Arreola-Risa C, Cameron P, Civil I, Grigoriou G, Gruen RL et al. Emergency Medical Service (EMS) systems in developed and developing countries. Injury International Journal of the Care of the Injured. 2007; 38: 1001-1013.
33. Reynolds TA, Stewart B, Drewett I, Salerno S, Sawe HR, Toroyan T, Mock C. The Impact of Trauma Care Systems in Low-and Middle-Income Countries. Annual Review of Public Health. 2017; 38: 507-532.
34. Razmkon A. Priorities and Concerns for Research on Neurotrauma in the Developing World. Bulletin of Emergency and Trauma. 2013; 1(1): 5-6.
35. Burton A. A key traumatic brain injury initiative in India. The Lancet Neurology. 2016; 15: 1011-1012.
36. Centre for Excellence for Early Childhood Development (CEED). Injury prevention. 2011. CEED/Strategic Knowledge Cluster on Early Child Development (SKC-ECD). Canada.
37. Arksey H, O'Malley L. Scoping studies: towards a methodological framework. International Journal of Social Research Methodology. 2005; 8(1): 19-32.
38. Callese TE, Richards CT, Shaw P, Schuetz SJ, Paladion L, Issa N, Swaroop M. Trauma system development in low-and middle-income countries: a review. Journal of Surgical Research. 2015; 193: 300-307.
39. Porchia BR, Baldasseroni A, Dellisanti C, Lorini C, Bonaccorsi G. Effectiveness of two interventions in preventing traffic accidents: a systematic review. Annali di igiene. 2014; 26: 63-75.
40. Zaal D. Traffic Law Enforcement: A Review of the Literature. 1994. Monash University Accident Research Centre. Monash, Australia. Report No. 53.
41. Lefio A, Bachelet VC, Jiminez-Paneque R, Gomolan P, Rivas K. A systematic review of the effectiveness of interventions to reduce motor vehicle crashes and their injuries among the general and working populations. Pan American Journal of Public Health. 2018; 42: 1-8.
42. Grodum Eskesen T, Stokholm Baekgaard J, Steinmetz J, Rasmussen LS. Initial use of supplementary oxygen for trauma patients: a systematic review. BMJ Open. 2018; 8:e020880.
43. Lamontagne M-E, Gagnon C, Allaire A-S, Noreau L. Effect of rehabilitation length of stay on outcomes in individuals with traumatic brain injury or spinal cord injury: a systematic review protocol. Systematic Reviews; 2013: 2-59.
44. Pickering A, Cooper K, Harnan S, Sutton A, Mason S, Nicholl J. Impact of pre-hospital transfer strategies in major trauma and head injury: Systematic review, meta-analysis and recommendations for study design. Journal of Trauma and Acute Care surgery. 2015; 78 (1): 164-177.

For peer review only - http://bmjopen.bmj.com/site/about/guidelines.xhtml

45. Von Elm E, Schoettker P, Henzi I, Osterwalder J, Walder B. Pre-hospital tracheal intubation in patients with traumatic brain injury: systematic review of current evidence. British Journal of Anesthesia. 2009; 103 (3): 371-386.
46. Alzahrani MB, Alansazi AM, Alsaghirat MAM, Alotaibi KM, Binmahfoz YKA, Altowairqi FM et al. Prehospital Tracheal Intubation versus Emergency Department Intubation for Trauma Patients. The Egyptian Journal of Hospital Medicine. 2018; 70 (6): 1039-1046.
47. Lundine JP. Memory Restoration Using Computerized Cognitive Training After Pediatric Traumatic Brain Injury: A Review of the Evidence. EBP Briefs. 2017; 12 (3): 1-17.
48. Stalder-Lüthy F, Messerli-Bürgy N, Hofer H, Frischknecht E, Znoj H, Barth J. Effect of Psychological Interventions on Depressive Symptoms in Long-Term Rehabilitation After an Acquired Brain Injury: A Systematic Review and Meta-Analysis. Archives of Physical Medicine and Rehabilitation. 2013; 94: 1386-1397.
49. Bunn F, Collier T, Frost C, Ker K, Roberts I, Wentz R. Traffic calming for the prevention of road traffic injuries: systematic review and meta-analysis. Injury prevention. 2003; 9: 200-204.
50. Newman ANL, Gravesande J, Rotella S, Wu SS, Topp-Nguyen N, Kho ME et al. Physiotherapy in the neurotrauma intensive care unit: A scoping review. Journal of Critical Care. 2018; 48: 390-406.

For peer review only

For peer review only - http://bmjopen.bmj.com/site/about/guidelines.xhtml

APPENDIX : SEARCH STRATEGY FOR MEDLINE 1946 to date: returned 18013 records

1. Brain Injuries, Traumatic/ or Craniocerebral Trauma/
2. (neurotrauma or “traumatic brain injur*” or “head injur*” or “acquired brain injur*” or “craniocerebral trauma”).ti,ab.
3. 1 or 2
4. Accidents, Traffic/
5. (“road traffic injur*” or “road traffic collision* or “road traffic accident*” or “road traffic crash*” or “road crash*” or “road accident*” or “road collision*” or “road injur*” or “traffic accident*” or “traffic collision*” or “traffic crash*” or “traffic injur*” or “motor vehicle accident*” or “motor vehicle collision*” or “motor vehicle crash*”). ti,ab.
6. 4 or 5
7. 3 or 6
8. Protective Devices/
9. Primary Prevention/
10. Secondary Prevention/
11. Tertiary Prevention/
12. Environment Design/
13. Law Enforcement/
14. Accident Prevention/or Safety/
15. Emergency Medical Services/
16. Health Services Accessibility/
17. Triage/
18. First Aid/
19. Oxygen/or Oxygen Inhalation Therapy/
20. Rehabilitation/or Neurological Rehabilitation/
21. Mindfulness/
22. (“trauma prevent*” or “accident prevent*” or “crash prevent*” or “injury prevent*” or “prevent* strategy*” or “prevent* intervention*” or “prevent* polic*” or “prevent* programme*” or “prevent* initiative*” or “primary prevent*” or “secondary prevent*” or “tertiary prevent*” or “protective device*” or “environmental design” or “traffic calming system” or “traffic law enforcement” or “law enforcement” or “traffic legislation” or legislation or “graduated driv* licen*” or “access to care” or “health service* accessibility” or “pre-hospital tracheal intubat*” or “pre-hospital transfer” or “pre-hospital triage” or triage or “first aid” or oxygen or “oxygen inhalation* therap*” or “pre-hospital resuscitati*” or “trauma system” or “emergency medical system” or “emergency medical service*” or safety or “road safety” or “traffic safety” or rehab* or “cognitive rehab*” or “psychological rehab*” or “physical rehab*” or “behavio?r* rehab*” or “neurological rehab*” or mindfulness).ti,ab.
23. Or/8-22
24. 7 and 23
25. Limit 24 to (english language and humans and yr=”1974-Current”)

For peer review only - http://bmjopen.bmj.com/site/about/guidelines.xhtml

BMJ Open

Mapping global evidence on strategies and interventions in neurotrauma and road traffic collisions prevention: a scoping review protocol

Journal:	*BMJ Open*
Manuscript ID	bmjopen-2019-031517.R1
Article Type:	Protocol
Date Submitted by the Author:	07-Oct-2019

Complete List of Authors:	M Selveindran, Santhani; University of Cambridge Institute of Public Health; University of Cambridge, NIHR Global Health Research Group on Neurotrauma<br>Khan, Muhammad; Northwest School of Medicine and Northwest General Hospital and Research Centre; University of Cambridge, NIHR Global Health Research Group on Neurotrauma<br>Simadibrata, Daniel; University of Indonesia; Newcastle University, Faculty of Medical Sciences<br>Hutchinson, Peter; Addenbrooke's Hospital, Department of Clinical Neurosciences; University of Cambridge, NIHR Global Health Research Group on Neurotrauma<br>Brayne, Carol; University of Cambridge Institute of Public Health; University of Cambridge, NIHR Global Health Research Group on Neurotrauma<br>Hill, Christine; University of Cambridge Institute of Public Health; University of Cambridge, NIHR Global Health Research Group on Neurotrauma<br>Kolias, Angelos; Addenbrooke's Hospital, Department of Clinical Neurosciences; University of Cambridge, NIHR Global Health Research Group on Neurotrauma<br>Joannides, Alexis; Addenbrooke's Hospital, Department of Clinical Neurosciences; University of Cambridge, NIHR Global Health Research Group on Neurotrauma<br>Servadei, Franco; Humanitas University and Research Hospital, Department of Neurosurgery; World Federation of Neurosurgical Societies<br>Rubiano Escobar, Andres ; Universidad El Bosque, Department of Neurosurgery; University of Cambridge, NIHR Global Health Research Group on Neurotrauma<br>Shabani, Hamisi; Muhimbili Orthopaedic Institute and Muhimbili University College of Allied Health Sciences; University of Cambridge, NIHR Global Health Research Group on Neurotrauma

**Primary Subject Heading**:	Global health
Secondary Subject Heading:	Public health
Keywords:	PUBLIC HEALTH, TRAUMA MANAGEMENT, PREVENTIVE MEDICINE

SCHOLARONE™
Manuscripts

TITLE: Mapping global evidence on strategies and interventions in neurotrauma and road traffic collisions prevention: a scoping review protocol

Santhani M Selveindran [1,2], Muhammad Mukhtar Khan [2,3], Daniel M Simadibrata [4,5], Peter JA Hutchinson [2,6], Carol Brayne [1,2], Christine Hill [1,2,7], Angelos Kolias [2,6], Alexis J Joannides [2,6], Franco Servadei [8,9], Andres M Rubiano [2,10], Hamisi K Shabani [2,11].

1. *Cambridge Institute of Public Health, University of Cambridge, Cambridge, UK.*
2. *NIHR Global Health Research Group on Neurotrauma, University of Cambridge, Cambridge, UK.*
3. *Northwest School of Medicine and Northwest General Hospital & Research Centre, Peshawar, Pakistan.*
4. *University of Indonesia, Depok, Jawa Barat, Indonesia.*
5. *Faculty of Medical Sciences, Newcastle University, Newcastle upon Tyne, UK.*
6. *Division of Neurosurgery, Department of Clinical Neurosciences, Addenbrooke's Hospital and University of Cambridge, Cambridge, UK.*
7. *Collaboration for Leadership in Applied Health Research and Care East of England (CLAHRC), Cambridge, UK.*
8. *Department of Neurosurgery, Humanitas University and Research Hospital, Milan, Italy*
9. *World Federation of Neurosurgical Societies, Nyon, Switzerland*
10. *Neuroscience Institute, El Bosque University, Bogotá, Colombia.*
11. *Neurological Surgery Unit, Muhimbili Orthopaedic Institute and Muhimbili University College of Allied Health Sciences, Dar es Salaam, Tanzania.*

Correspondence to: Dr Santhani M Selveindran, Cambridge Institute of Public Health, University of Cambridge School of Clinical Medicine, Forvie Site, Cambridge Biomedical Campus, Cambridge CB2 0SR, UK. Email: ss2604@medschl.cam.ac.uk. Tel: +44122330301.

Keywords: *Neurotrauma prevention, Road traffic collisions prevention, Preventative strategies, Preventative interventions, Low and middle income countries, High income countries*

Word count: 3172

For peer review only

ABSTRACT

Introduction: Neurotrauma is an important global health problem. This 'silent epidemic' is a major cause of death and disability in adolescents and young adults, with significant societal and economic impacts. Globally, the largest cause of neurotrauma is road traffic collisions (RTCs). Neurotrauma and RTCs are largely preventable, and many preventative strategies and interventions have been established and implemented over the last decades, particularly in high income countries. However, these approaches may not be applicable globally, due to variations in environment, resources, population, culture and infrastructure. This paper outlines the protocol for a scoping review which seeks not only to map the evidence on strategies and interventions in neurotrauma and RTCs prevention globally, but also to ascertain contextual factors that influence their implementation.

Methods and analysis: This scoping review will use the established methodology by Arksey and O'Malley. Eligible studies will be identified from five electronic databases (MEDLINE, EMBASE, CINAHL, Global Health/EBSCO and Cochrane Database of Systematic Reviews) and grey literature sources. We will also carry out bibliographic and citation searching of included studies. A two-stage selection process which involves screening of titles and abstracts, followed by full-text screening will be used to determine eligible studies which will undergo data abstraction using a customized, piloted data-extraction sheet. The extracted data will be presented using evidence mapping and a narrative summary.

Ethics and dissemination: Ethical approval is not required for this scoping review, which is the first step in a multiphase public health research project on the global prevention of neurotrauma. The final review will be submitted for publication to a scientific journal, and results will be presented at appropriate conferences, workshops and meetings.

Protocol registered on 5th April 2019 with Open Science Framework (https://osf.io/s4zk3/).

ARTICLE SUMMARY

STRENGTHS AND LIMITATIONS OF THIS STUDY

- This will be the first scoping review to identify and quantify the evidence on all types of preventative strategies and interventions for primary, secondary and tertiary prevention of neurotrauma, using all types of literature from both high and low and middle income countries.
- The scoping review utilises a well-established, rigorous scoping review methodology with a systematic, iterative search strategy.
- The literature search will be comprehensive, encompassing electronic databases with peer-reviewed literature, as well as a wide range of grey literature sources including government and non-governmental websites.
- A limitation of this review is the language restriction to English, which may result in the potential to miss relevant articles, especially in the grey literature.

For peer review only - http://bmjopen.bmj.com/site/about/guidelines.xhtml

INTRODUCTION

Neurotrauma is an important global health problem (1-2). Previous studies and systematic reviews, including one from the World Health Organization (WHO), estimate that the annual global incidence of neurotrauma is approximately 500 to 800 per 100,000 (3-4). This 'silent epidemic' continues to increase and is predicted to surpass many diseases as a major cause of death and disability (5). Indeed, WHO Global Burden of Disease estimates indicate that neurotrauma accounts for about 11.8 percent of total global disability-adjusted life years (DALYs) (3).

Although neurotrauma typically refers to injury to the brain and/or spinal cord, for this review, neurotrauma will focus on injuries to the head alone.

Globally, the largest cause of neurotrauma is road traffic collisions (RTCs) (3, 5). RTCs are the eighth leading cause of death in the world, and recent WHO analyses estimate that this could become the fifth leading cause of death by 2030 (6). RTCs are particularly common in low and middle income countries (LMICs) as a result of rapid urbanisation and motorisation with few or no safeguarding measures (6-8).

Neurotrauma, whether due to RTCs or other causes, poses major medical, public health and societal problems worldwide due to the impact it has on individuals, families, communities, governments and healthcare systems. (9-13).

Despite some country-to-country variability, many studies have shown that neurotrauma is more common in children and young adults (5, 10, 14). Neurotrauma is a major cause of death and the leading cause of disability in those under forty years of age, and is predicted to be the 3rd leading cause of premature death across all ages by 2020 (4,14). Generally, there is a bimodal age-specific neurotrauma incidence with peaks in childhood (0-10 years) and late adolescence/early adulthood (15-36 years) (10, 14). In terms of gender, the incidence of neurotrauma is consistently higher in males with ratios ranging from 1.6 to 6.5, particularly in adolescence and early adulthood (4-5, 14).

The demographic impact of neurotrauma in itself has societal and economic implications. As neurotrauma death is highest in males in the age-group where most would be the breadwinners within their families, this can result in financial loss or burden (9). In those who survive, a large proportion would require prolonged hospitalisation, long rehabilitation and suffer long-term disabilities (15). Deficits associated with neurotrauma are cognitive (impaired attention, poor executive functioning, poor decision-making), physical (impaired motor functioning), behavioural (aggressive behaviour, impulsivity) and emotional (depression, anxiety) (16-18). All this would not only affect their ability to work, but to care for themselves and to participate in society, impacting the lives of relatives and the community around them (9, 19-20). There would be loss of quality of life (intangible costs), not only for the sufferer, but also for their caregivers (12, 18).

Economic losses do not only affect families, but also have impact on countries and the world as a whole. The global economic cost of neurotrauma is estimated to be in the region of USD 400 billion annually, where most of the aggregate cost of neurotrauma takes the form of indirect costs, resulting from the adverse effect on people's ability to work (loss of productivity) (9, 12, 21). The direct costs, which refer to all resources consumed within the health-care sector as a result of neurotrauma, pose a burden on healthcare systems (9, 22).

The management of neurotrauma encompasses pre-hospital care (lasting for minutes to hours), in-hospital (hours to weeks) and post-acute care (weeks to years) (12, 14, 23-24). Pre-hospital care involves the correct assessment and efficient treatment at the site of injury or during transfer, and

For peer review only - http://bmjopen.bmj.com/site/about/guidelines.xhtml

the prompt transport to a formal, appropriately equipped healthcare facility (12, 24). In-hospital care would be the surgical and non-surgical treatment of neurotrauma, including imaging, neuro-monitoring and intensive care, whereas post-acute care refers to any form of rehabilitative interventions to enable and empower patients to have an increased quality of life, as neurotrauma is a life-long disorder (5, 23-24).

The lifetime costs of medical treatment for neurotrauma is said to range from USD 600,000 to USD 1.8 million per case and are almost always borne by the healthcare system (21-22). In addition to financial constraint, the high burden of neurotrauma may be disproportionate to the resources available for managing the patient load, particularly in LMICs where there are deficiencies in facilities and manpower (5, 14, 25).

Therefore, neurotrauma prevention would be more beneficial in that it would not only save lives, but reduce prevalence of disabilities and save costs within and outside the health-care system (12). Neurotrauma prevention is not only limited to measures which target injury occurrence (primary prevention), but also involves providing adequate medical response to manage and minimise harm following an injury (secondary prevention), and mitigating the sequelae and reducing consequent disability (tertiary prevention) (12, 26-27). These can be applied at societal, community, household and individual levels (12, 28).

Many high income countries (HICs) have implemented multiple intervention strategies and projects that have contributed significantly to the reduction of neurotrauma (7). These range from adapting the environment, legislation, safety education and skills training, to strengthening post-trauma response systems and improving access to acute and post-acute care (12, 23, 29-31). However, these strategies are not often seen in LMICs due to differences in environment, resources, population, culture and infrastructure (32-34). It is therefore necessary that strategies and interventions match contexts in order to effectively reduce the burden of neurotrauma, not only in LMICs but globally.

The objective of this scoping review is to identify and quantify the breadth of evidence on neurotrauma preventative strategies and interventions, provide a descriptive overview of what these are, where they are implemented, and to ascertain contextual factors that influence their implementation. This in turn will be used to inform policy and practice in the area of neurotrauma prevention. As RTCs are a major cause of neurotrauma, this review will delve into RTCs prevention, in addition to neurotrauma prevention as a whole. RTCs will be defined as a collision or incident involving at least one motorised or unmotorised (i.e. pedestrian, cyclist) vehicle in motion, on a road to which the public has right of access (35).

This methodology is appropriate for reviewing a large body of literature to generate an overview of the evidence on this subject, summarise the results and identify research gaps (36). To date, several reviews have been carried out to identify, examine, and study effectiveness of specific preventative strategies and interventions in particular regions or countries (7, 32, 37-48). There is also a scoping review on interventions to reduce road traffic injuries, but this was limited to the African continent, and another one specifically on physiotherapy after neurotrauma (6, 49). The value of this scoping review is that it will include all types of evidence on all types of preventative strategies and interventions in both HICs and LMICs.

For peer review only - http://bmjopen.bmj.com/site/about/guidelines.xhtml

METHODS

Protocol design

This scoping review is informed by the framework proposed by Arksey and O'Malley (2005). This is a six step framework that includes:

1. Identifying the research question
2. Identifying relevant studies
3. Study selection
4. Charting the data
5. Collating, summarising, and reporting the results
6. Consulting and translating knowledge

As this scoping review is part of a wider scoping exercise that includes stakeholder consultation, stage 6 will not be carried out.

Stage 1: Identifying the research question

The overarching research question, given the purpose of this review is "What are the global strategies and interventions in neurotrauma and RTCs prevention?" Being part of a wider scoping exercise that seeks to understand contextual issues relating to neurotrauma and RTCs prevention, the following sub-review questions were identified:

1. What are the strategies and interventions in neurotrauma and RTCs prevention in LMICs?
2. What are the strategies and interventions in neurotrauma and RTCs prevention in HICs?
3. In what settings are these strategies and interventions carried out (i.e school-based/community-based)?
4. What are the contextual factors that can affect or influence the implementation of these strategies and interventions?

Stage 2: Identifying relevant studies

In this stage, we will identify the criteria to be used to select studies that will be included in the review. The Population, Concept and Context (PCC) mnemonic will be used to determine study eligibility, as follows (50):

Population: Adults and children, where children will be taken as those below the age of 18 years.

Concept: Any strategy and/or intervention implemented for the prevention of neurotrauma or RTCs. For this review, this would include primary prevention, referring to any measures that eliminate neurotrauma or RTCs; secondary prevention, which will refer to pre-hospital care systems and any interventions delivered as part of this care; and tertiary prevention, which will be any form of rehabilitative interventions for neurotrauma patients. We will include publications which describe established preventative strategies and interventions. Studies reporting on intervention outcomes which relate to neurotrauma and/or RTCs prevention, or on factors influencing implementation will also be included. For RTCs prevention, we will include studies on strategies and interventions that prevent crashes and those that prevent neurotrauma, should a crash occur.

We will exclude the following studies: Papers on strategies and interventions that do not specifically prevent neurotrauma and/or RTCs, studies where strategies and interventions are ill-defined, publications describing proposed strategies or interventions, or opinions on an intervention, studies on strategies and interventions without any specific context, papers describing only the prevalence

For peer review only - http://bmjopen.bmj.com/site/about/guidelines.xhtml

of intervention utilisation without measuring outcomes or exploring factors influencing utilisation, and publications discussing in-hospital management or care of neurotrauma patients.

For publications on rehabilitative strategies and interventions (tertiary prevention), only articles discussing specific approaches that address cognitive, physical, behavioural and emotional rehabilitation will be included. Articles including pharmacological interventions will be excluded.

Context: The preventative strategies and interventions would be carried out or delivered in any country (LMIC or HIC) and in any setting (i.e. community or health-care facility).

The following criteria will also be utilised for study eligibility:

Language: Only studies written in English will be included.

Time frame: Searches will begin from 1974 onwards, which was when the World Health Assembly adopted Resolution WHA 27.59, calling for Member States to address the major public health problem of RTCs (30). For databases established after 1974, searches will commence from their inception.

Types of studies: As a scoping review is designed to cover a wide spectrum of literature, there will be no restrictions as to the type of studies that will be included in this review.

The following electronic databases will be searched:

1. MEDLINE (Ovid)
2. EMBASE (Ovid)
3. CINAHL (EBSCO host)
4. Global Health (EBSCO host)
5. Cochrane Database of Systematic Reviews

An academic librarian was consulted and gave suggestions on key concepts, and text-word and Medical Subject Headings searching. The initial search terms were determined by the main author where the concepts 'Neurotrauma', 'Road traffic collisions' and 'Prevention' were each expressed with a list of synonyms. This was supplemented with keywords and phrases from relevant articles retrieved from an exploratory search of MEDLINE and Google Scholar (see online supplementary material, **APPENDIX**, which details the search strategy developed for MEDLINE). The search strategy will be utilised in the other electronic databases, where the search terms will be adapted and modified based on the requirement of the individual database. As scoping review searches are quite iterative, additional search terms may be identified and incorporated into the search strategy.

We will also search grey literature to identify any non-indexed papers. This would include web-based data sources such as Open Grey, Prevention Information and Evidence Library, The Grey Literature Report and The National Institute for Health and Care Excellence (NICE).

Where possible, we will search for unpublished studies from websites of governmental, non-governmental or patient-based organisations engaged in neurotrauma or RTCs prevention (i.e. Global Alliance of NGOs for Road Safety).

Bibliographies of eligible studies will be examined to identify any original studies not obtained through the searches. Additionally, citation searching of these studies, using Google Scholar will also be carried out.

Search results will be imported to a bibliographic manager which will be used to store references and remove duplicates. Where necessary, duplicates will also be removed manually.

For peer review only - http://bmjopen.bmj.com/site/about/guidelines.xhtml

Stage 3: Study selection

The screening for potentially eligible publications will follow a two-stage process:

Stage 1: The titles and abstract of potentially eligible studies will be screened against selection criteria, and will be categorised as 'include', 'exclude' or 'uncertain'.

Stage 2: Full-text articles will be sourced for those publications under the 'include' and 'uncertain' categories. The full-text articles will be assessed against the selection criteria and the reason for exclusion will be documented.

This process will be carried out by two reviewers independently. Any disagreements regarding publication inclusion will be resolved through discussion between the two reviewers or third party adjudication.

The process of study selection will be reported using the Preferred Reporting Items for Systematic Reviews and Meta-Analyses (PRISMA) flow chart.

For peer review only

Stage 4: Charting the data

The extraction of data for a scoping review is known as 'charting the data' and should present information about the study that aligns to the objective and questions of the review.

Appropriate data from the eligible studies will be extracted manually using a customised data-extraction sheet designed using Microsoft ® Word. This will be in tabular form and will be piloted on several studies to ensure all relevant results are extracted, pertinent to the research questions. If necessary, the categories will be modified and the extraction sheet revised.

In line with the research questions for this review, the following data will be extracted:

Publication detail:

- Citation
- Publication type (published/unpublished)
- Publication source (journal/website)
- Country of origin
- Funding source

Study characteristics:

- Study design/type
- Aim(s)/objective(s) of study
- Study location/setting

If Systematic review/meta-analyses:

- Number of studies included
- Years of publication of included studies
- Countries where included studies were conducted

For peer review only - http://bmjopen.bmj.com/site/about/guidelines.xhtml

Participant characteristics:

- Age/age-range
- Gender
- Ethnicity
- Occupation/socio-economic status

Preventative strategy/intervention

- Description of intervention
- Delivery of intervention (how and by whom)
- Setting of intervention
- Length and intensity of intervention

Reported outcome(s)

Factors influencing outcome(s)

The charting of data is an iterative process and the extraction fields may change depending on the included studies.

Stage 5: Collating, summarising and reporting results

The data collected will be presented using two strategies:

1. Evidence mapping to provide a comprehensive and concise descriptive map of the breadth of research on neurotrauma or RTCs prevention. The extracted data will be categorised based on the year of publication, study design, geographic region, type of participants, type of prevention (primary, secondary, tertiary) and type of intervention. Data will be presented in tabular and graphical forms.

2. A narrative summary describing the included studies and how the results relate to the review objective and research questions.

The exact reporting format cannot be determined until the data is charted, which would depend on the literature found. If possible, a specific framework will be sought to categorise and map the findings. However, the results will be reported using the Preferred Reporting Items for Systematic Reviews and Meta-Analysis: Extension for Scoping Reviews (PRISMA-ScR) (51).

There will be no assessment of quality as it is beyond the scope of the review.

For peer review only - http://bmjopen.bmj.com/site/about/guidelines.xhtml

Patient and Public Involvement

Although patients and public are not involved in the design and conduct of this scoping review, it forms part of a larger scoping exercise which seeks to gather contextual issues and align research priorities. The review will be used to guide questioning during the stakeholder consultation, which is the second component of this exercise. Stakeholders will include policy makers, law enforcement officials, healthcare staff, and members of patient and advocacy groups, who could have been neurotrauma patients. Comparisons will be made between findings from this review and the stakeholder consultation.

This review will also form part of the report on the scoping exercise that will be sent for publication and shared with relevant stakeholders and research funders. We will aim to utilise government and non-governmental websites and facilities to disseminate data to patients and members of the public.

CONCLUSION

This article presents a protocol for a scoping review, which is a comprehensive, rigorous and transparent methodology. This review, which includes both peer reviewed and grey literature, will contribute to research in the area of neurotrauma and RTCs prevention by identifying the breadth of preventative strategies and interventions, their contexts and factors influencing implementation. Through this research, policy and practice can be informed, leading to the development and implementation of evidence-based, context-appropriate, feasible strategies and interventions in resource constrained settings. This review also allows for discerning gaps in knowledge, and to provide recommendations for future research.

This scoping review is the first step in a multiphase research project which aims to study the epidemiology of neurotrauma caused by RTCs in LMICs and to understand factors amenable to its prevention in these countries.

ETHICS AND DISSEMINATION

As the scoping review methodology entails collecting, reviewing and synthesising material from publicly available publications, no ethical approval is necessary. The final review will be submitted for publication to a scientific journal. Where possible, results will be presented at appropriate conferences, workshops and meetings, both nationally and internationally.

For peer review only

STATEMENTS

Acknowledgements: The authors thank Dr Veronica Phillips for giving advice and feedback on the search strategy. They also thank Dr Sarah Kelly for providing advice on the research questions and overall conduct of the scoping review.

Authors' contributions: SMS conceived the idea of a scoping review, developed the research questions and methods, and contributed substantially to drafting and editing this protocol. MMK and DMS helped conceptualise the study inclusion and exclusion criteria. PJAH, AK, CB, CH, AJJ, FS, AMR and HKS gave guidance on study design and provided advisory contributions to the development of this protocol. All authors give approval to the publishing of the protocol manuscript.

Funding: This research was commissioned by the National Institute of Health Research using Official Development Assistance (ODA) funding (Grant number: RG89187). The views expressed in this publication are those of the author(s) and not necessarily those of the NHS, National Institute for Health Research or the Department of Health.

Competing interests: None

Provenance and peer review: Not commissioned; externally peer reviewed

Open Access: This is an open access article distributed in accordance with the Creative Commons Attribution Non Commercial (CC BY-NC 4.0) license which permits others to distribute, remix, adapt, build upon this work non-commercially, and license their derivative works on different terms, provided the original work is properly cited, appropriate credit is given, any changes made indicated, and the use is non-commercial. See: http://creativecommons.org/licenses/by-nc/4.0/

REFERENCES

1. Green SE, Bosch M, McKenzie JE, O'Connor DA, Tavender EJ, Bragge P et al. Improving the care of people with traumatic brain injury through the Neurotrauma Evidence Translation (NET) program: protocol for a program of research. Implementation Science. 2012; 7 (24). Available from http://www.implementationscience.com/content/7/1/74
2. GBD 2016 Traumatic Brain Injury and Spinal Cord Injury Collaborators. Global, regional, and national burden of traumatic brain injury and spinal cord injury, 1990-2016: a systematic analysis for the Global Burden of Disease Study 2016. Lancet Neurology. 2019; 18: 56-87.
3. Dewan M, Rattani A, Gupta S, Baticulon RE, Hung Y, Punchak M et al. Estimating the global incidence of traumatic brain injury. Journal of Neurosurgery. April 27, 2018. **DOI:** https://doi.org/10.3171/2017.10.JNS17352.
4. Feigin VL, Barker-Collon S, Krishnamurthi R, Theadom A, Starkey N. Epidemiology of ischaemic stroke and traumatic brain injury. Best Practice and Research Clinical Anaesthesiology. 2010; 24: 485-494.
5. Hyder AA, Wunderlich CA, Puvanachandra P, Gururaj G and Kobusingye OC. The impact of traumatic brain injuries: A global perspective. NeuroRehabilitation. 2007; 22(5): 341-353.
6. Bonnet E, Lechat L, Ridde V. What interventions are required to reduce road traffic injuries in Africa? A scoping review of the literature. PLoS One. 2018; 13 (11): e208195.

For peer review only - http://bmjopen.bmj.com/site/about/guidelines.xhtml

7. Staton C, Vissoci J, Gong E, Toomey N, Wafula R, Amdelgadir J et al. Road Traffic Injury Prevention Initiatives: A Systematic Review and Metasummary of Effectiveness in Low and Middle Income Countries. PLoS ONE. 2015; 11(1): e0144971.
8. Johnson WD, Griswold DP. Traumatic Brain Injury: a global challenge. The Lancet Neurology Commission. 2017; 16: 948-950.
9. Majdan M, Plamcikova D, Brazinova A, Rusnak M, Nieboer D, Feigin V et al. Epidemiology of traumatic brain injuries in Europe: cross-sectional analysis. Lancet Public Health. 2016; 1: e76-83.
10. Saatian MR, Ahmadpoor J, Mohammadi Y, Mazloumi E. Epidemiology and Pattern of Traumatic Brain Injury in a Developing Country, Regional Trauma Center. Bulletin of Emergency Trauma. 2018; 6 (1): 45-53.
11. Wilson MH. Traumatic Brain Injury: an underappreciated public health issue. The Lancet. 2016; 1(2): pe44.
12. Maas AIR, Menon DK, Adelson PD, Andelic N, Bell MJ. Belli A, Bragge P, Brazinova A et al. Traumatic brain injury: integrated approaches to improve prevention, clinical care, and research. The Lancet Neurology Commission. 2017; 16: 987-1048.
13. Wright DW, Kellermen A, McGuire LC, Chen B. Popovic T. CDC Grand Rounds: Reducing Severe Traumatic Brain Injury in the United States. Morbidity and Mortality Weekly Report. 2013; 62 (27): 549-552.
14. Iaccariono C, Carretta A, Nocolosi F, Morselli C. Epidemiology of severe traumatic brain injury. Journal of Neurosurgical Sciences. 2018; 62 (5): 535-541.
15. Stocchetti N, Zainer ER. Chronic impact of traumatic brain injury on outcome and quality of life: a narrative review. Critical care. 2016; 20 (148). DOI.10.1186/s13054-016-1318-1.
16. Rubiano AM, Carney N, Chestnut R, Puyana JC. Global neurotrauma research challenges and opportunities. Nature. 2015; 527: S193-S197.
17. Ponsford J, Spitz G. MERRC-TAC Linkage Report: identifying different patterns of outcome and their predictors. 2016. Monash-Epworth Rehabilitation Research Centre, Monash University, Australia: Research report 150-0816-R02.
18. Connolly D, O'Dowd T. The Impact of the Different Disabilities arising from Head Injury on the Primary Caregiver. British Journal of Occupational Therapy. 64 (1): 41-45.
19. Access Economics Pty Limited. The economic cost of spinal cord injury and traumatic brain injury in Australia. 2009. The Victorian Neurotrauma Initiative, Victoria, Australia.
20. Juengst SB, Arenth PM, Raina KD, McCue M, Skidmore ER. Affective State and Community Integration after Traumatic Brain Injury. American Journal of Physiotherapy and Medical Rehabilitation. 2014; 93 (12): 1086-1094.
21. Parsonage M. The costs of traumatic brain injury. February 2017. Centre for Mental Health. London, United Kingdom.
22. Fu TS, Jing R, McFaull SR, Cusimano MD. Health and Economic Burden of Traumatic Brain Injury in the Emergency Department. The Canadian Journal of Neurological Sciences Inc. 2016; 43 (2). 238-247.
23. Cnossen MC, Lingsma HF, Tenovuo O, Maas AIR, Menon D et al. Rehabilitation After Traumatic Brain Injury: A Survey in 70 European Neurotrauma Centres Participating in the CENTER-TBI Study. Journal of Rehabilitation Medicine. 2017; 49: 395-401.
24. Dash HH, Chavali S. Management of traumatic brain injury patients. Korean Journal of Anesthesiology. 2018; 71(1): 12-21.
25. Zia N, Mehmood A, Namaganda RH, Ssenyonjo H, Kobusingye O, Hyder AA. Causes and outcomes of traumatic brain injuries in Uganda: analysis from a pilot hospital registry. Trauma Surgery and Acute Care Open. 2019; 4: e000259.

For peer review only - http://bmjopen.bmj.com/site/about/guidelines.xhtml

26. Hoz S, Moscote-Salazar LR. Prevention of Neurotrauma: An Evolving Matter. Journal of Neurosciences in Rural Practice. 2017; 8 (Suppl 1): S141-S143.
27. Gururaj G, Kolliri SVR, Chandramouli BA, Subbakirshna DK, Kraus JF. Traumatic Brain Injury. National Institute of Mental Health and Neuro Sciences. 2005. Publication no.61. Bangalore.
28. Wright DW, Kellermen A, McGuire LC, Chen B. Popovic T. CDC Grand Rounds: Reducing Severe Traumatic Brain Injury in the United States. Morbidity and Mortality Weekly Report. 2013; 62 (27): 549-552.
29. Wood S, Bellis MA, Watkins S. Road traffic accidents: A review of evidence for prevention from the UK focal point for violence and injury prevention. 2010. Centre for Public Health, Liverpool.
30. World Health Organization (WHO). World Report on Road Traffic Injury Prevention. 2004 WHO. Geneva.
31. Roudsari BS, Nathens AB, Arreola-Risa C, Cameron P, Civil I, Grigoriou G, Gruen RL et al. Emergency Medical Service (EMS) systems in developed and developing countries. Injury International Journal of the Care of the Injured. 2007; 38: 1001-1013.
32. Reynolds TA, Stewart B, Drewett I, Salerno S, Sawe HR, Toroyan T, Mock C. The Impact of Trauma Care Systems in Low-and Middle-Income Countries. Annual Review of Public Health. 2017; 38: 507-532.
33. Razmkon A. Priorities and Concerns for Research on Neurotrauma in the Developing World. Bulletin of Emergency and Trauma. 2013; 1(1): 5-6.
34. Burton A. A key traumatic brain injury initiative in India. The Lancet Neurology. 2016; 15: 1011-1012.
35. Centre for Excellence for Early Childhood Development (CEED). Injury prevention. 2011. CEED/Strategic Knowledge Cluster on Early Child Development (SKC-ECD). Canada.
36. Arksey H, O'Malley L. Scoping studies: towards a methodological framework. International Journal of Social Research Methodology. 2005; 8(1): 19-32.
37. Callese TE, Richards CT, Shaw P, Schuetz SJ, Paladion L, Issa N, Swaroop M. Trauma system development in low-and middle-income countries: a review. Journal of Surgical Research. 2015; 193: 300-307.
38. Porchia BR, Baldasseroni A, Dellisanti C, Lorini C, Bonaccorsi G. Effectiveness of two interventions in preventing traffic accidents: a systematic review. Annali di igiene. 2014; 26: 63-75.
39. Zaal D. Traffic Law Enforcement: A Review of the Literature. 1994. Monash University Accident Research Centre. Monash, Australia. Report No. 53.
40. Lefio A, Bachelet VC, Jiminez-Paneque R, Gomolan P, Rivas K. A systematic review of the effectiveness of interventions to reduce motor vehicle crashes and their injuries among the general and working populations. Pan American Journal of Public Health. 2018; 42: 1-8.
41. Grodum Eskesen T, Stokholm Baekgaard J, Steinmetz J, Rasmussen LS. Initial use of supplementary oxygen for trauma patients: a systematic review. BMJ Open. 2018; 8:e020880.
42. Lamontagne M-E, Gagnon C, Allaire A-S, Noreau L. Effect of rehabilitation length of stay on outcomes in individuals with traumatic brain injury or spinal cord injury: a systematic review protocol. Systematic Reviews; 2013: 2-59.
43. Pickering A, Cooper K, Harnan S, Sutton A, Mason S, Nicholl J. Impact of pre-hospital transfer strategies in major trauma and head injury: Systematic review, meta-analysis and recommendations for study design. Journal of Trauma and Acute Care surgery. 2015; 78 (1): 164-177.

For peer review only - http://bmjopen.bmj.com/site/about/guidelines.xhtml

44. Von Elm E, Schoettker P, Henzi I, Osterwalder J, Walder B. Pre-hospital tracheal intubation in patients with traumatic brain injury: systematic review of current evidence. British Journal of Anaesthesia. 2009; 103 (3): 371-386.
45. Alzahrani MB, Alansazi AM, Alsaghirat MAM, Alotaibi KM, Binmahfoz YKA, Altowairqi FM et al. Prehospital Tracheal Intubation versus Emergency Department Intubation for Trauma Patients. The Egyptian Journal of Hospital Medicine. 2018; 70 (6): 1039-1046.
46. Lundine JP. Memory Restoration Using Computerized Cognitive Training After Pediatric Traumatic Brain Injury: A Review of the Evidence. EBP Briefs. 2017; 12 (3): 1-17.
47. Stalder-Lüthy F, Messerli-Bürgy N, Hofer H, Frischknecht E, Znoj H, Barth J. Effect of Psychological Interventions on Depressive Symptoms in Long-Term Rehabilitation After an Acquired Brain Injury: A Systematic Review and Meta-Analysis. Archives of Physical Medicine and Rehabilitation. 2013; 94: 1386-1397.
48. Bunn F, Collier T, Frost C, Ker K, Roberts I, Wentz R. Traffic calming for the prevention of road traffic injuries: systematic review and meta-analysis. Injury prevention. 2003; 9: 200-204.
49. Newman ANL, Gravesande J, Rotella S, Wu SS, Topp-Nguyen N, Kho ME et al. Physiotherapy in the neurotrauma intensive care unit: A scoping review. Journal of Critical Care. 2018; 48: 390-406.
50. The Joanna Briggs Institute. Joanna Briggs Institute Reviewers' Manual 2015 edition/supplement. 2015. The Joanna Briggs Institute: Adelaide.
51. Tricco AC, Lillie E, Zarin W, O'Brien KK, Colquhoun H et al. PRISMA Extension for Scoping Reviews (PRISMA-ScR): Checklist and Explanation. Annals of Internal Medicine. 2018; 169: 467-473.

For peer review only

For peer review only - http://bmjopen.bmj.com/site/about/guidelines.xhtml

APPENDIX : SEARCH STRATEGY FOR MEDLINE 1946 to date: returned 18013 records

1. Brain Injuries, Traumatic/ or Craniocerebral Trauma/
2. (neurotrauma or "traumatic brain injur*" or "head injur*" or "acquired brain injur*" or "craniocerebral trauma").ti,ab.
3. 1 or 2
4. Accidents, Traffic/
5. ("road traffic injur*" or "road traffic collision* or "road traffic accident*" or "road traffic crash*" or "road crash*" or "road accident*" or "road collision*" or "road injur*" or "traffic accident*" or "traffic collision*" or "traffic crash*" or "traffic injur*" or "motor vehicle accident*" or "motor vehicle collision*" or "motor vehicle crash*"). ti,ab.
6. 4 or 5
7. 3 or 6
8. Protective Devices/
9. Primary Prevention/
10. Secondary Prevention/
11. Tertiary Prevention/
12. Environment Design/
13. Law Enforcement/
14. Accident Prevention/or Safety/
15. Emergency Medical Services/
16. Health Services Accessibility/
17. Triage/
18. First Aid/
19. Oxygen/or Oxygen Inhalation Therapy/
20. Rehabilitation/or Neurological Rehabilitation/
21. Mindfulness/
22. ("trauma prevent*" or "accident prevent*" or "crash prevent*" or "injury prevent*" or "prevent* strategy*" or "prevent* intervention*" or "prevent* polic*" or "prevent* programme*" or "prevent* initiative*" or "primary prevent*" or "secondary prevent*" or "tertiary prevent*" or "protective device*" or "environmental design" or "traffic calming system" or "traffic law enforcement" or "law enforcement" or "traffic legislation" or legislation or "graduated driv* licen*" or "access to care" or "health service* accessibility" or "pre-hospital tracheal intubat*" or "pre-hospital transfer" or "pre-hospital triage" or triage or "first aid" or oxygen or "oxygen inhalation* therap*" or "pre-hospital resuscitati*" or "trauma system" or "emergency medical system" or "emergency medical service*" or safety or "road safety" or "traffic safety" or rehab* or "cognitive rehab*" or "psychological rehab*" or "physical rehab*" or "behavio?r* rehab*" or "neurological rehab*" or mindfulness).ti,ab.
23. Or/8-22
24. 7 and 23
25. Limit 24 to (english language and humans and yr="1974-Current")

For peer review only - http://bmjopen.bmj.com/site/about/guidelines.xhtml
